# NOD-like receptors mediate inflammatory lung injury during plateau hypoxia exposure

**DOI:** 10.1186/s40101-020-00242-w

**Published:** 2020-10-07

**Authors:** Haiyan Wang, Xue Lin, Xiaoyan Pu

**Affiliations:** 1 College of Medicine, Qinghai University, Xining, 810001 Qinghai Province China; 2 Qinghai Normal University, Xining, 810007 Qinghai Province China

**Keywords:** Plateau hypoxia, Lung, Inflammatory, Transcriptome sequence, NOD-like receptors

## Abstract

**Background:**

The lung is an important target organ for hypoxia treatment, and hypoxia can induce several diseases in the body.

**Methods:**

We performed transcriptome sequencing for the lungs of rats exposed to plateau hypoxia at 0 day and 28 days. Sequencing libraries were constructed, and enrichment analysis of the differentially expressed genes (DEGs) was implemented using the Gene Ontology (GO) and Kyoto Encyclopedia of Genes and Genomes (KEGG). Subsequently, experimental validation was executed by quantitative real-time PCR (qRT-PCR) and western blot.

**Results:**

The results showed that the nucleotide-binding oligomerization domain (NOD)-like receptor (NLR) signaling pathway that was involved in immunity may play a crucial function in lung injury caused by plateau hypoxia. And the expressions of *NOD1*, *NOD2*, *IL-1β*, *TNF-α*, *IL-6*, and *IL-18* were higher at 28 days of exposure to plateau hypoxia than that at 0 day. Similarly, CARD9, MYD88, p38 MAPK, and NF-κB p65, which are related to the NF-κB and MAPK signaling pathways, also demonstrated increased expression at 28 days exposure to plateau hypoxia than at 0 day.

**Conclusions:**

Our study suggested that the NF­κBp65 and p38 MAPK signaling pathways may be activated in the lungs of rats during plateau hypoxia. Upregulated expression of NF­κBp65 and p38 MAPK can promote the transcription of downstream inflammatory factors, thereby aggravating the occurrence and development of lung tissue remodeling.

## Introduction

About 2% of the world’s total population lives in high-altitude areas (generally ≥ 2500 m), and in China alone, areas above 3000 m account for 25% of the country’s land [1]. Moreover, with the continuous development of tourism, the economy, and national defense, increasing numbers of people will enter high-altitude areas to live and work [2]. The plateau is an ecological environment which possesses some special features, such as low oxygen, high radiation, and low pressure [3]. When humans access the plateau environment from the plains, their organs and tissues undergo physiological hypoxia. High altitude actuates the body to decrease partial pressure and oxygen saturation of the arterial blood, eventually leading to tissue hypoxia [4]. On the other hand, hypoxia can cause high-altitude pulmonary edema (HAPE) [5], high-altitude cerebral edema (HACE) [6], polycythemia, and cardiovascular diseases [7]. As the lung is the most sensitive organ to oxygen, hypoxia can cause lung damage. Especially concerning is the risk that acute high-altitude hypoxia or mild altitude reactions can result in HAPE [5].

As with all pattern-recognition receptors (PRRs), NLRs are located intracellularly [8, 9] and recognize pathogen-associated molecular patterns (PAMPs) [10] to defend the host from invading bacteria, viruses, and other pathogens. There are 22 NLRs in humans and at least 33 NLRs in mice. The majority of NLRs comprise a variable N-terminal effector domain, a central NOD, and a C-terminal leucine-rich repeat (LRR) domain [8]. The NLR family includes five subfamilies according to the type of effector domain, which is either a caspase recruitment domain (CARD), a baculoviral inhibitor of apoptosis protein repeat (BIR) domain, or a pyrin domain (PYD) [11]. Among the five subfamilies, the NLRC subfamily is comprised of the CARD-containing molecules NOD1, NOD2, and NLRC3–5 [11]. The earliest discovered NLRs [12, 13], NOD1, and NOD2 are both expressed in the lungs [14–20]. After NOD1 and NOD2 recognize the PAMPs, the nuclear factor (NF)-κB and MAP kinase (MAPK) signaling pathways are activated through Rip2 kinase. Subsequently, pro-inflammatory cytokines, such as TNFα, IL-1β, IL-6, and IL-18, chemokines, and antimicrobial peptides are activated and secreted to blossom an effective immune response. Typically, severe inflammatory responses are found in HAPE patients [21]. Therefore, we speculate that the NOD-like receptor signaling pathway may be tightly involved with the occurrence and development of HAPE.

Our previous results revealed increased alveolar compensation or increased alveolar capillaries by Hematoxylin and Eosin (HE) staining when the lung had been exposed to plateau hypoxia for 28 days (unpublished data). However, the molecular mechanism of damage to the lungs of rats exposed to plateau hypoxia is still vague. In this present study, transcriptome sequencing methods were employed to research the effect of plateau hypoxia on rat lungs. Additionally, based on the transcriptome analysis results, we verified the expression of certain genes and proteins related to the NF-κB and MAPK signaling pathways by the quantitative real-time PCR (qRT-PCR) and western blot, respectively. These data present a new avenue to study the molecular regulation mechanism of plateau hypoxia on the lungs.

## Materials and methods

### Animals

Twelve specific-pathogen-free (SPF) rats, weighing 200 ± 20 g, were bought from the Medical Laboratory of Xi’an Jiaotong University, experimental animal license number: SCXK (shan) 2018-001. The temperature of the rearing room was 25 ± 2°C, and the relative humidity was 40 to 60%. All rats were fed a standard diet and had free drinking water. Twelve rats were randomly divided into two groups of six rats each:28-day group and 0 day group. In the 28-day group: These six rats were raised in Maduo County, Qinghai Province, at an altitude of 4200 m for 28 days to collect the lung tissues. In the 0-day group: These six rats were housed at the Medical Laboratory of Xi’an Jiaotong University at an altitude of 500 m and then sacrificed at day 0 in this study. Therein, three rats of each group were used for RNA extracts, which was divided into two parts for mRNA-seq and qRT-PCR analysis, respectively. Another three rats were used for western blot analysis. All animals were used in accordance with all animal welfare laws, and the study was approved by the Ethical Committee of Qinghai University.

### RNA-seq library preparation and Illumina sequencing

The total RNA from the lungs of rats exposed to plateau hypoxia at 0 days and 28 days was extracted using an Animal Total RNA Isolation Kit (RE-03014, FOREGENE, Chengdu, China) in accordance with the manufacturer’s instructions. The purity and integrity of the obtained RNA were also quantified by an Agilent BioAnalyzer 2100. Each RNA sample was both used for transcriptome sequencing and experimental validation through qRT-PCR. The RNA sample from one individual at 0 day or 28 days was collected to generate one replicate sample. Equal amounts of RNA from the 0-day and 28-day samples were employed for the construction of mRNA-Seq libraries. An Illumina TruSeq RNA Sample Prep Kit was used to construct the mRNA-Seq libraries at Beijing Novogene Zhiyuan Technology Co., Ltd. (Beijing, China). In brief, the poly-T oligo-attached magnetic beads were employed to purify mRNA from total RNA. Subsequently, the purified mRNA was fragmented and reversely transcribed into cDNA. Then, connect the adaptor to the cDNA molecule and amplify the fragment by PCR. The Illumina HiSeq 2500 sequencing platform was used to execute the sequencing in paired-end reads (2 × 101 bp). The FastQC method was used to evaluate the quality of data from mRNA sequencing (http://www.bioinformatics.babraham.ac.uk/projects/fastqc/).

### Bioinformatics analysis of mRNA-seq data

The mRNA-Seq reads generated by the Illumina HiSeq platform. Subsequently, the adaptor sequences and the low-quality (< 20) bases at the 5′ and 3′ ends were removed by Trimmomatic (v0.30) [22]. The clean reads longer than 70 bp were employed for further experiment. The reads were mapped to the *Rattus norvegicus* genome (Ensembl RGSC3.4) using TopHat v1.4.1 [23] with default parameters (*-r 400 -p 8*) after preprocessing of mRNA-Seq data. The gene abundances were quantified using the RSEM (https://www.biostat.wisc.edu/~cdewey/). The readings were normalized via DEseq2 v. 1.24.0 (http://bioconductor.org/packages/stats/bioc/DESeq2/) [24] and was used for differential expression analysis of read counts. The following criteria are used here: Default parameters: Benjamini & Hochberg (BH) *p* adjust < 0.05 &|log_2_FC| ≥ 1 after multiple comparisons. The resulting *p* values were adjusted through BH false discovery rate (FDR) algorithm. Differential expression of genes was significant only when the FDR values were < 0.05 (controlling the expected FDR to no more than 5%) and the log_2_FoldChange (FC) was ≥ 1. This tool employs a negative binomial distribution model to detect for differential gene expression. Ami GO with the default parameters were employed to acquire gene ontology terms of each gene and analyze GO functional enrichment via hypergeometric tests with FDR correction to gain an adjusted *p* value between particular test gene groups and the whole annotation data set, severally. The DEGs in Kyoto Encyclopedia of Genes and Genomes (KEGG) pathway were analyzed using Cytoscape with the ClueGO plugin [25, 26].

### cDNA synthesis and real-time PCR analysis of gene expression

cDNA synthesis was performed using a PrimeScript RT reagent kit (RR047A, Takara, Japan) following the manufacturer’s instructions. qRT-PCR was performed using the A PIKORed 96 (ThermoFisher, USA) with primers (listed in Table 1) and using the TB Green TM Premix Ex TaqTM II (Tli RNaseH Plus) (Takara, RR820A) as described previously [27]. The expression of each gene was first normalized to that of β-actin and was presented as a fold change by calculating the average expression level of each of the three samples divided by that of the controls at the same time point.

**Table 1:** Primers used in this study

Primer name	Forward primer (5′–3′)	Reverse primer (5′–3′)
β-actin	GAAGATCAAGATCATTGCTCC	TACTCCTGCTTGCTTGCGATCCA
IL-1	ATCCTCTCCAGTCAGGCTTCCTTGTG	AGCTCTTGTCGAGATGCTGCTGTGA
IL-2	TGTTGCTGGACTTACAGGTGCTCCT	CCACCACAGTTGCTGGCTCATCATC
IL-6	ACAGAGGATACCACCCACAACAGACC	CGGAACTCCAGAAGACCAGAGCAGAT
IL-18	TGCCTGATATCGACCGAACAGCCAAC	ACAGATAGGGTCACAGCCAGTCCTCT
NOD1	CTCAAAGGAGGACCTGCTGCTGGA	GAAGACAGTCTCGCCATGCTCGTTGA
NOD2	GGCAGCACAGGTGGACTCTGAGGATA	GCAGCAGCCTTAGCAGCAGTGAGTT
TNT-α	CCAGCAGGAGGGAGAACAGCAACT	CCGCCACGAGCAGGAATGAGAAGAG

### SDS-PAGE and western blot analysis

The total protein was extracted from rat lung samples, and the protein concentration was quantified using the BCA protein quantification kit. SDS-PAGE and western blot analyses were executed as the previous study [28]. In brief, the proteins were separated by 12% SDS-PAGE and then were electrically transferred to a PVDF membrane. The PVDF membrane was incubated with the primary antibody (the primary antibody and the corresponding dilution concentration are listed in Table 2) at 4 °C overnight after pre-blocking with TBST (containing 5% skim milk (Anchor, New Zealand)) for 2 h at room temperature. The membrane was incubated with goat-anti-rabbit IgG (H&L)-HRP or goat-anti-mouse IgG (H&L)-HRP (Abcam, Cambridge, UK) diluted 1:5000 in TBST (containing 5% skim milk) for 2–3 h at room temperature after three washes with TBST. The reaction was visualized using an ECL Luminescence Kit (affinity, KF001) for 1 min. Scan analysis was performed using a gel image analysis imaging system, and the results were expressed as the relative expression of the target protein. The relative expression of the target protein = integrated optical density value (IOD) of the target protein/integrated optical density value (IOD) of the internal reference.

**Table 2:** The primary antibody and the corresponding dilution concentration

Reagent or resource	Source	Identifier	Dilution concentration
Rabbit polyclonal anti-MYD88	Abcam	Cat# ab2064	1:1000
Rabbit polyclonal anti-P-P65	Abcam	Cat# ab86299	1:1000
Mouse polyclonal anti-CARD9	Abcam	Cat# ab169623	1:1000
Mouse polyclonal anti-CD86	Abcam	Cat# ab220188	1:1000
Rabbit polyclonal anti-P-P38	Abcam	Cat# ab4822	1:1000
Rabbit polyclonal anti-β-actin	Abcam	Cat# ab8227	1:2000
Goat Anti-Mouse IgG H&L	Abcam	Cat# ab6789	1:5000
Goat Anti-Rabbit IgG H&L	Abcam	Cat# ab6721	1:5000

### Statistical analysis

The unpaired *t* test was used to examine the significance of the difference of mean values between two groups using the SPSS 19.0 package (SPSS Inc. Chicago, IL, USA). All results were recorded as the means ± SE, and the differences were considered statistically non-significant, significant, or extremely significant when *p* ≥ 0.05, *p* < 0.05, and *p* < 0.01, respectively.

## Results

### Characterization of the transcriptome sequence

**Fig. 1:**
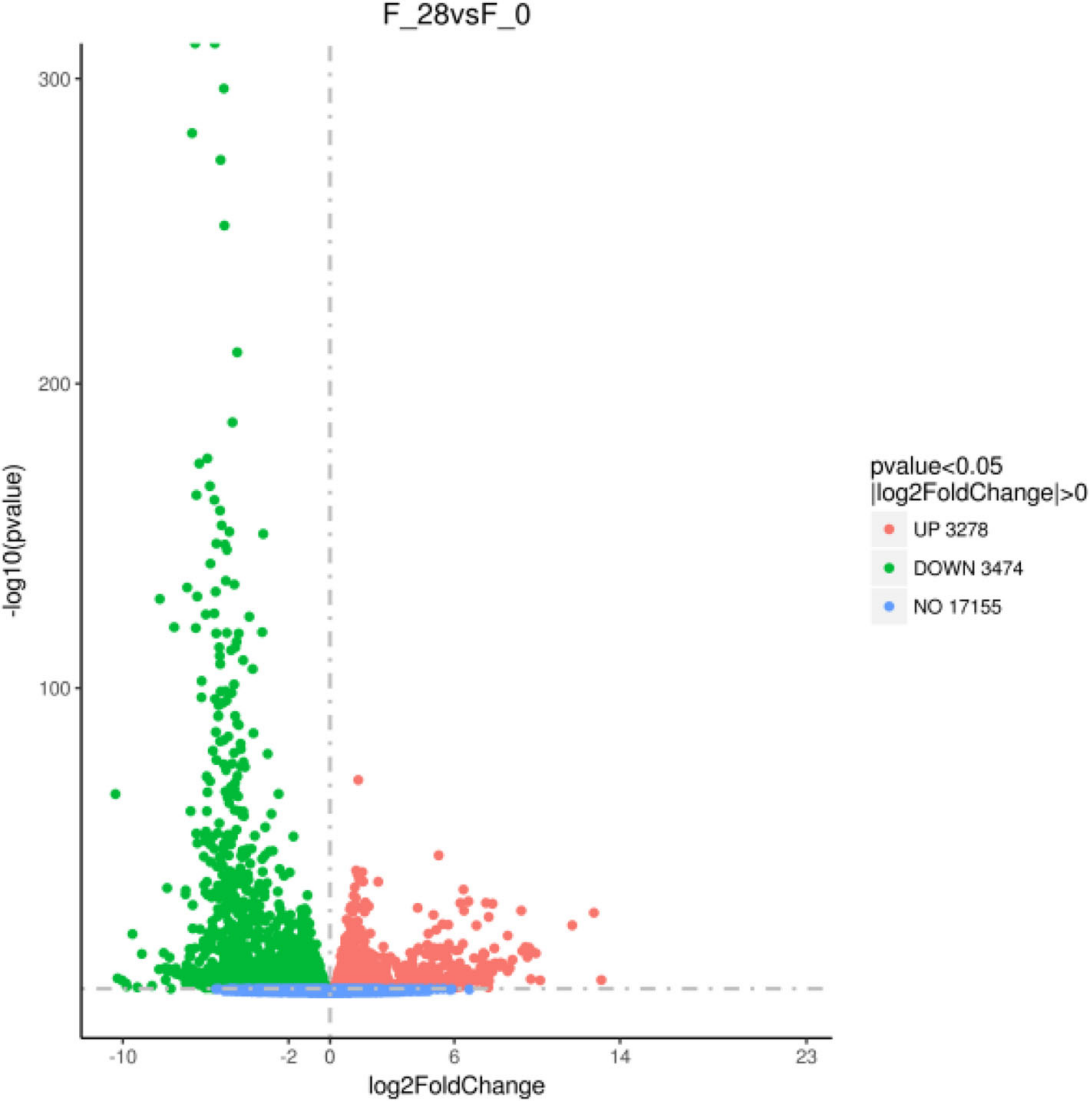
A Volcano plot of genes identified between 0 day and 28 days

To explore the molecular mechanism of lung damage after exposure to plateau hypoxia, three replicates of the samples at 0 day or 28 days were sequenced, and the transcriptome libraries were constructed. A total of 42.46 M and 58.20 M raw reads were generated for the 0-day and 28-day samples on average, respectively. Likewise, after the low-quality reads and adapter sequences were filtered out of the raw data, the clean reads were 41.58 M and 56.50 M on average for the 0-day and 28-day groups, respectively. The clean read ratio values were 97.93% and 97.09%, the Q30% values of the clean reads were 93.25% and 93.14%, and the GC values were 48.86% and 50.35% at 0 day and 28 days on average, respectively (Table 3). The uniquely mapped clean reads were utilized in gene expression analysis based on FPKM. After exposure to plateau hypoxia for 28 days, 3474 downregulated and 3278 upregulated genes were present compared to gene expression at 0 day (Fig. 1). The top 100 upregulated and downregulated DEGs (|log_2_FC| ≥ 1 and FDR < 0.05) were listed in supplementary files Tables S1 and S2.

**Table 3:** Summary of the reads and mapping results

Sample	0–1 days	0–2 days	0–3 days	0-day mean	28–1 day	28–2 days	28–3 days	28-day mean
Total raw reads (M)	39.91	44.84	42.63	42.46	61.40	57.87	55.32	58.20
Total clean reads (M)	39.22	43.79	41.72	41.58	59.69	56.06	53.75	56.50
Total clean bases (Gb)	5.88	6.57	6.26	6.24	8.95	8.41	8.06	8.47
Clean reads Q20 (%)	97.91	97.75	96.72	97.46	97.35	97.79	97.31	97.48
Clean reads Q30 (%)	94.19	93.86	91.69	93.25	92.82	93.82	92.77	93.14
Clean reads ratio (%)	98.28	97.65	97.85	97.93	97.22	96.88	97.16	97.09
Total mapping (%)	93.10	92.94	92.37	92.80	95.05	94.34	94.7	94.70
Multiple mapping (%)	4.6	4.91	4.51	4.67	5.6	5.98	5.56	5.71
Unique mapping (%)	88.51	88.04	87.86	88.14	89.44	88.37	89.14	88.98
GC (%)	48.66	49.18	48.75	48.86	50.36	50.54	50.14	50.35

### GO enrichment analysis of differentially expressed genes after plateau hypoxia exposure at 0 day and 28 days

**Fig. 2:**
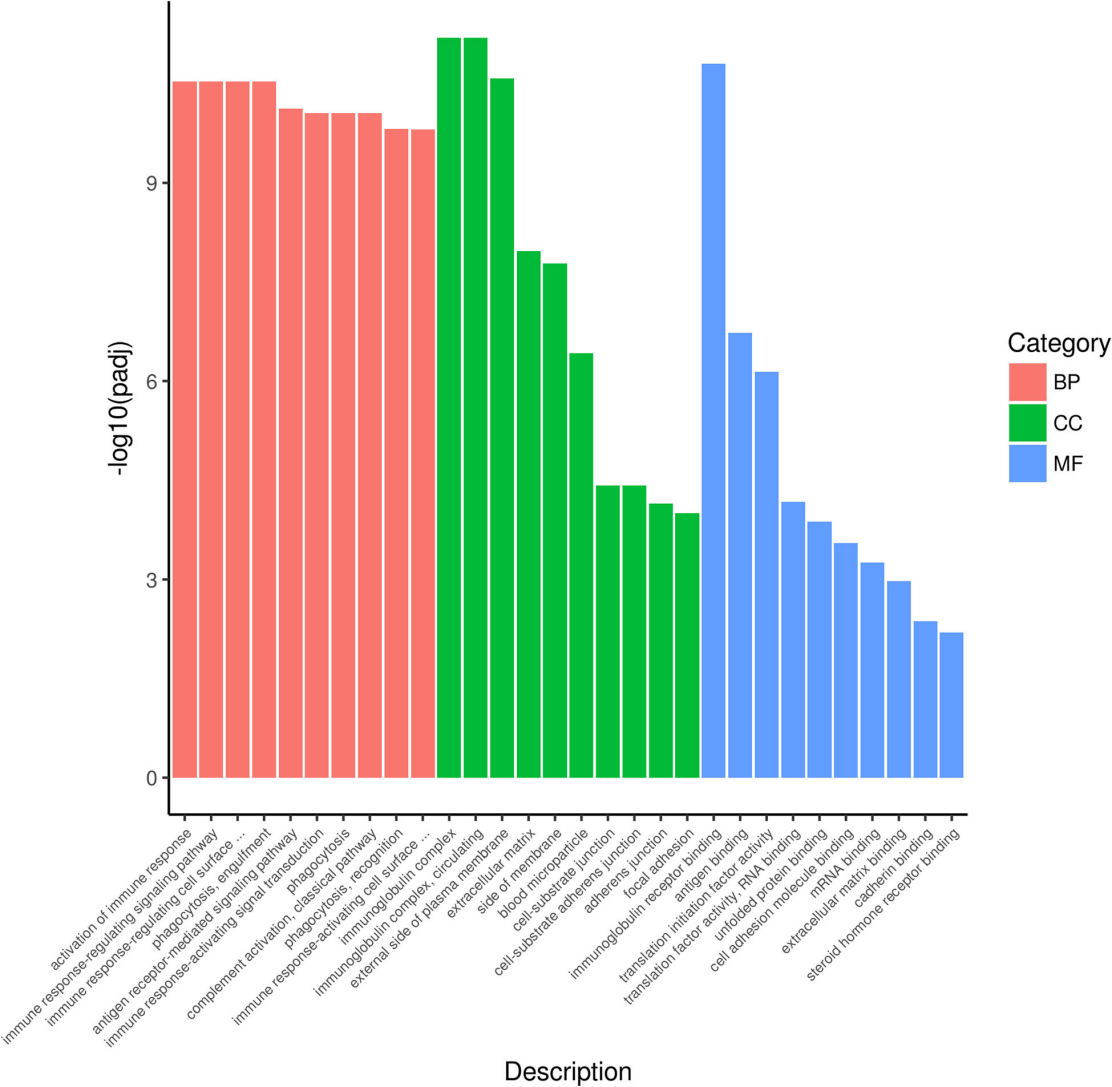
GO enrichment analysis of lung DEGs in the rats exposed to plateau hypoxia at 0 day and 28 days

To further explore the molecular mechanism of lung injury after exposure to plateau hypoxia, DEGs were subjected to GO enrichment analysis relay for the following three categories: biological processes, cellular components, and molecular function (Fig. 2). The results showed that most genes in all three aforementioned categories were involved in the immune process, such as the activation of the immune response and the immune response-regulating signaling pathway at the biological process level, immunoglobulin complex circulating at the cellular components level, and immunoglobulin receptor binding and antigen binding at the molecular function level. In brief, GO enrichment analysis suggested that immunity may play an important role in lung injuries caused by plateau hypoxia.

### KEGG enrichment analysis of differentially expressed genes after plateau hypoxia exposure at 0 day and 28 days

**Fig. 3:**
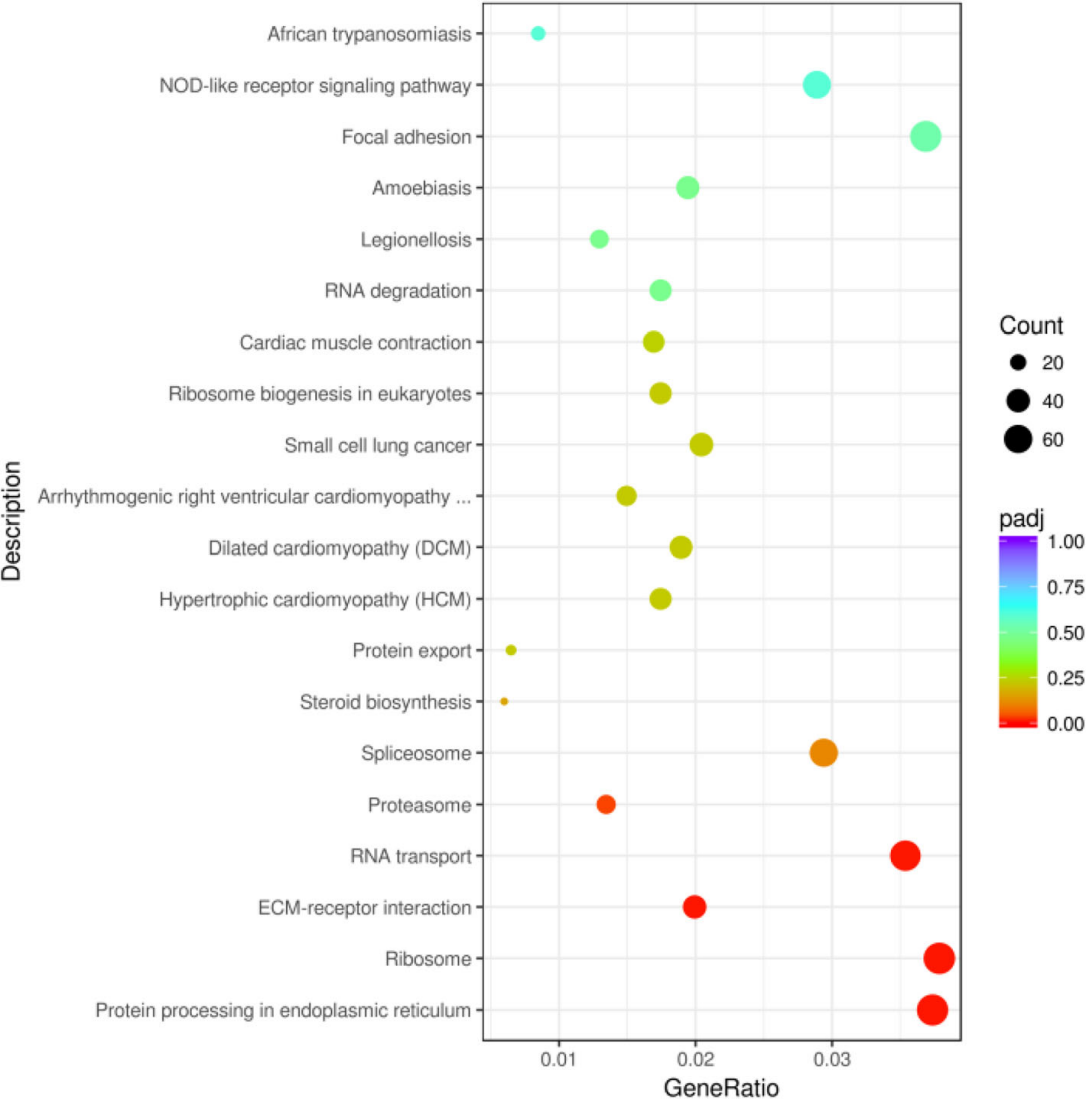
KEGG enrichment analysis of lung DEGs in the rats exposed to plateau hypoxia at 0 day and 28 days

**Fig. 4:**
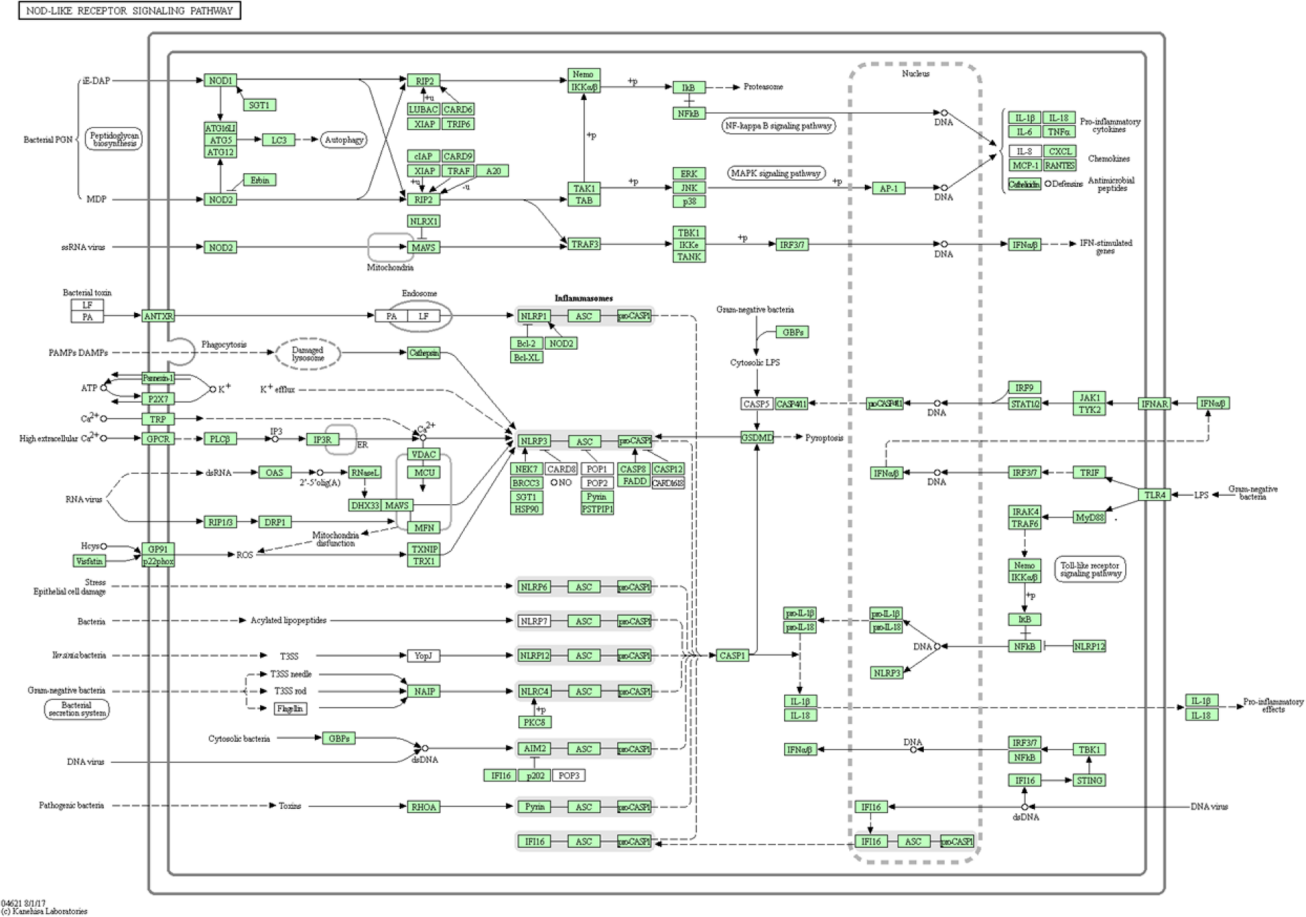
KEGG enrichment map of NOD-like receptor signaling pathway

To acknowledge the role of immunity in lung injuries caused by plateau hypoxia, KEGG enrichment analysis was carried out on top 20 most enriched pathways of DEGs (Fig. 3). Enriched KEGG pathways from the DEGs are listed in supplementary files Table S3. Among the many enriched signaling pathways, the NOD-like receptor signaling pathway involved in immunity was significantly upregulated, which suggested that the NOD-like receptor signaling pathway may play a crucial function in lung injury caused by plateau hypoxia. The KEGG enrichment map plots of NOD-like receptor signaling pathway were shown in Fig. 4, and 46 upregulated DEGs and 11 downregulated DEGs were involved in NOD-like receptor signaling pathway (listed in supplementary files Table S4).

### Gene expression of the lung exposed to plateau hypoxia at 0 day and 28 days

**Fig. 5:**
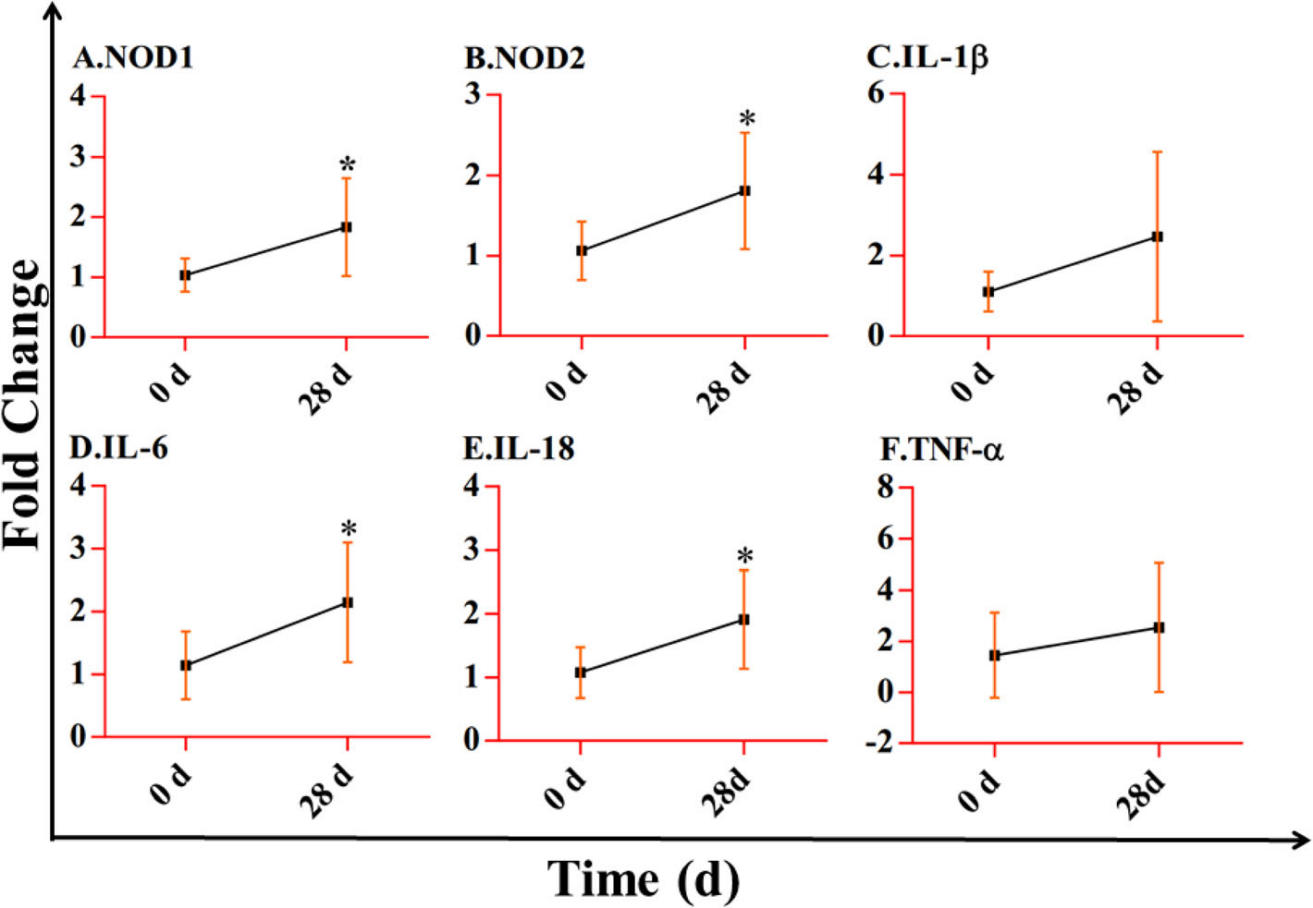
The expression of *NOD1*, *NOD2*, *IL-1β*, *TNF-α*, *IL-6*, and *IL-18* in the lungs of rats exposed to plateau hypoxia at 0 day and 28 days

Based on the transcriptome sequence analysis, the mRNA expression of six genes related to the NOD-like receptor signaling pathway was detected using qRT-PCR. As both *NOD1* and *NOD2* were expressed in the lungs of rats, we detected the expression of *NOD1* and *NOD2* after exposure to plateau hypoxia at 0 day and 28 days. We found that both *NOD1* and *NOD2* expression were significantly higher at a 28-day exposure than that at 0 day (Fig. 5a, b).

Similarly, some inflammatory factors were expressed after plateau hypoxia exposure. *IL-1β* and *TNF-α* expression remained higher at 28 days than at 0 day of plateau hypoxia exposure (Fig. 4c, f). Importantly, the expression of *IL-6* and *IL-18* was significantly higher at 28 days than that at 0 day of plateau hypoxia exposure (Fig. 5d, e).

### Protein expression of the lung at 0 day and 28 days of plateau hypoxia exposure

**Fig. 6:**
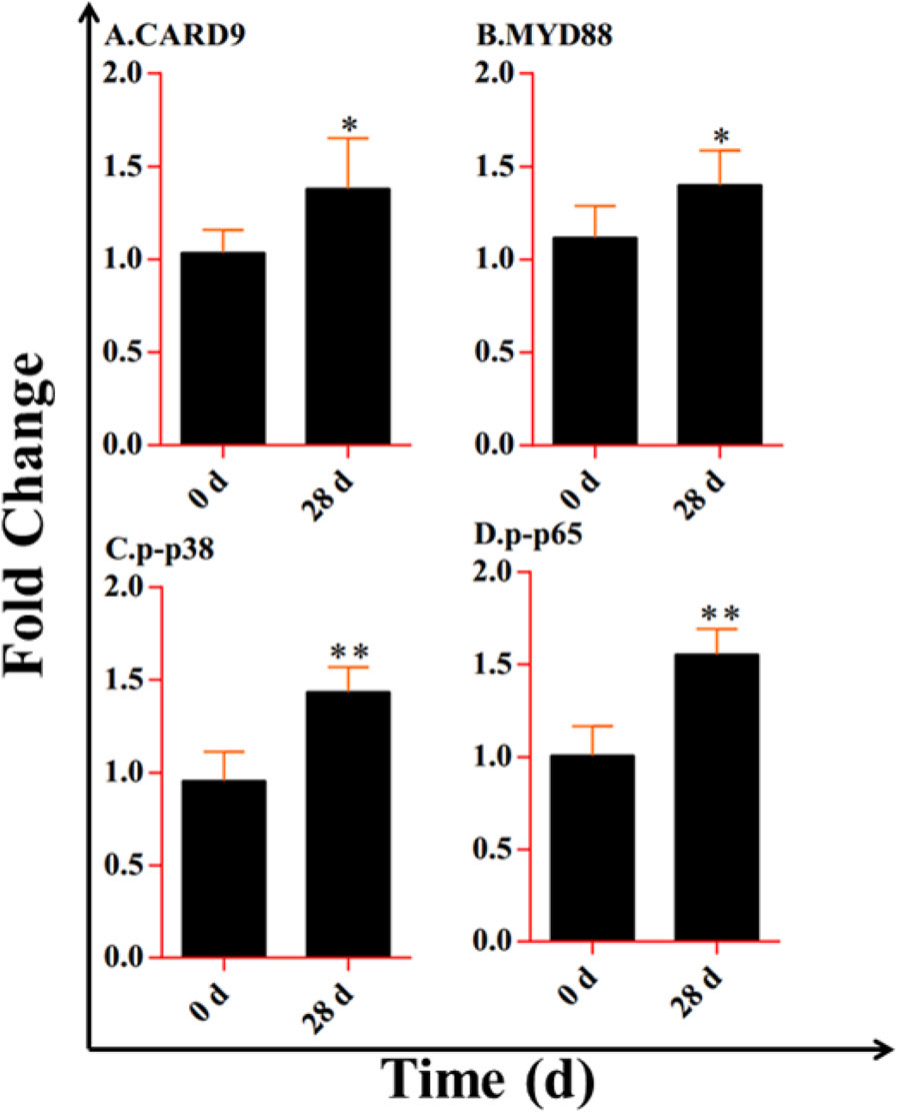
The expression of CARD9, MYD88, p-p38, and p-p65 in the lungs of rats exposed to plateau hypoxia at 0 day and 28 day

**Fig. 7:**
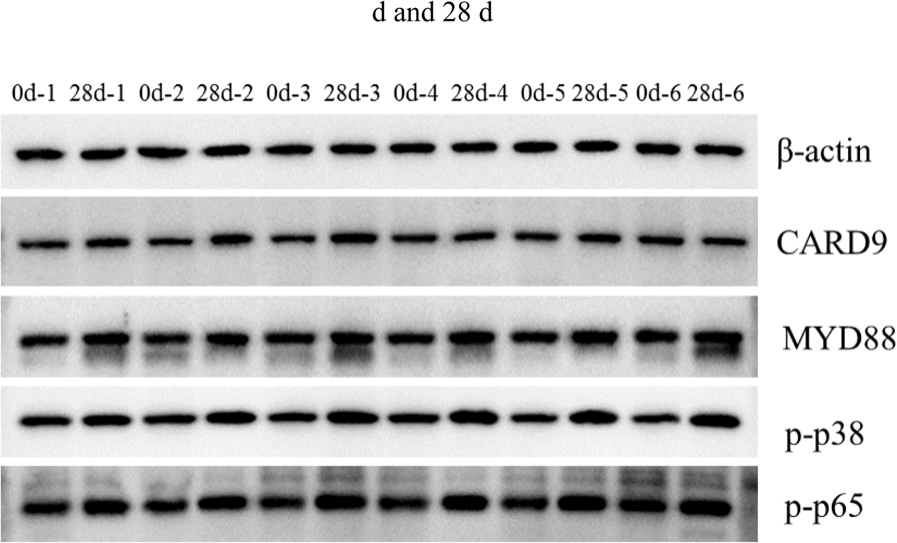
Western blot analysis of CARD9, MYD88, p-p38, and p-p65 in the lungs of rats exposed to plateau hypoxia at 0 day and 28 days

Furthermore, the expression of proteins associated in the NOD-like receptor signaling pathway was examined using western blot. As NLRs can activate the NF-κB and MAPK signaling pathways, we detected the expression of some key proteins related to these two pathways. Upon recognition of PAMPs, NOD recruits adaptor proteins MyD88 and CARD9 to activate the NF-κB and MAPK pathways. Thus, we detected the protein expression of MyD88 and CARD9. The results showed that the expression of CARD9 and MYD88 was significantly higher at 28 days than that at 0 day of plateau hypoxia exposure. Since activation of the p38 MAPK signaling pathway can further directly activating the NF-κB p65 pathway, we found that the expression of phosphorylated p38 MAPK and NF-κBp65 was extremely significantly higher at 28 days than that at 0 day (Figs. 6 and 7). In brief, these results indicated that the NF-κB and MAPK pathways are activated when the rats are exposed to plateau hypoxia.

## Discussion

The lung is the most sensitive organ to hypoxia. When the partial pressure of oxygen in the alveoli decreases to a certain threshold, rapid pulmonary artery contraction may occur, and corresponding changes in the bronchi and alveolar cells in the lung will also occur. In this present study, the transcriptome analysis obtained from GO and KEGG enrichment analysis of the DEGs showed that the NOD-like receptor signaling pathway involved in immunity may play an important role in lung injury caused by plateau hypoxia. Further experimental validation suggested that the NF­κBp65 and p38 MAPK signaling pathways were activated in the lungs of rats during plateau hypoxia. Upregulated expression of NF­κBp65 and p38 MAPK can promote the transcription of downstream inflammatory factors, thereby aggravating the occurrence and development of lung tissue remodeling.

With the increasing progress of transcriptomic technology, lung transcriptome sequencing has been studied extensively. The transcriptome sequence of the lung in mice infected with influenza A virus [29] and that of the PRRSV infection in porcine lungs [30] have been analyzed. Meanwhile, other research focuses on transcriptome analysis of sick or cancerous lungs [31–33]. Additionally, the transcriptome analysis of lung after treated with adenocarcinoma drugs has also gained attention [34, 35]. After mice were infected with influenza A virus, the expression of virus-induced chemokines, pro-inflammatory cytokines, adhesion molecules, and inflammatory cells and inflammatory enzymes, antibodies and complement activation were upregulated. Meanwhile, the transcriptome analysis of lung after treated with adenocarcinoma drugs revealed that majority of the identified genes were enriched in the PI3K/AKT, actin cytoskeleton regulation, mitogen-activated protein kinase, and focal adhesion pathways [34]. In the present study, we executed the transcriptome analysis as in a previous study of transcriptome analysis of the yak distributed in the Qinghai-Tibetan Plateau that the biological process analysis showed that those involved in immunological cells accounted for the highest ratio [36]. The experimental validation with qRT-PCR and western blot was utilized to obtain results suggesting that the NOD-like receptors mediate inflammatory lung injury after plateau hypoxia exposure.

Generally, NOD1 and NOD2 were both found in the lung. NOD1 has been found in endothelial cells, human airway smooth muscle cells, lung epithelial cells, and different types of leukocytes [14–17], while NOD2 is expressed in neutrophils, bronchial epithelial cells, and alveolar macrophages [18–20]. Thus, we analyzed the expression of *NOD1* and *NOD2* after the rats were exposed to plateau hypoxia. After PAMPs recognize NOD genes both the NF-κB and MAPK pathways were activated. Subsequently, certain inflammatory cytokines and chemokines such as IL-1β, TNF-α, IL-6, and IL-18, which are pivotal for stimulation and recruitment of additional effector cells, were secreted to develop an effective immune response [15, 37–39]. Hence, we determined the expression of these genes above. As major regulators of inflammation and immunity, IL-1β, TNF-α, IL-6, and IL-18 play important roles in initiating inflammatory reactions [40, 41]. The results suggested that the expression levels of the examined genes, including *NOD1*, *NOD2*, *IL-1β*, *TNF-α*, *IL-6,* and *IL-1*8, were all increased after exposure to plateau hypoxia. These results indicated that plateau hypoxia may cause an inflammatory reaction in the lungs of rats.

Additionally, upon recognition of their respective PAMPs, NOD recruits adaptor proteins such as MyD88 and CARD9 to activate the NF-κB and MAPK pathways [42]. It has been shown that p38 MAPK participates in the activation of NF-κB. Activation of the p38 MAPK signaling pathway can promote IκBα double phosphorylation and degradation, thereby directly activating the NF-κB p65 pathway, suggesting that p38 MAPK may participate in the pathological damage of lung tissue by activating NF-κB p65 [43]. Our results also revealed that the expression levels of the examined proteins, including CARD9, MyD88, p38 MAPK, and NF-κB p65, were all increased after exposure to plateau hypoxia, indicating that the NF-κB and MAPK pathways are activated when the rats are exposed to plateau hypoxia.

In summary, the present study demonstrated that NOD-like receptors can mediate inflammation while the NF­κBp65 and p38 MAPK signaling pathways may be activated in the lungs of rats during plateau hypoxia. Upregulated expression of NF­κBp65 and p38 MAPK can promote the transcription of downstream inflammatory factors, thereby aggravating the occurrence and development of lung tissue remodeling. Our research provides a novel insight into the molecular mechanism of lung injury in rats after plateau hypoxia exposure.

## Supplementary information

**Additional file 1: Table S1.** List of top 100 upregulated DEGs expressed between 0 d and 28 d.

**Additional file 2: Table S2.** List of top 100 downregulated DEGs expressed between 0 d and 28 d.

**Additional file 3: Table S3.** Enriched KEGG pathways from DEGs between 0 d and 28 d.

**Additional file 4: Table S4.** The detail information of DEGs derived from NOD-like receptor signaling pathway.

## Data Availability

The datasets used and/or analyzed during the current study are available from the corresponding author upon reasonable request.

## Abbreviations

DEGs: The differentially expressed genes
GO: Gene Ontology
KEGG: Kyoto Encyclopedia of Genes and Genomes
qRT-PCR: Real-time PCR
NLR: Nucleotide-binding oligomerization domain-like receptor
HAPE: High-altitude pulmonary edema
HACE: High-altitude cerebral edema
PRRs: Pattern-recognition receptors
PAMPs: Pathogen-associated molecular patterns
CARD: Caspase recruitment domain
NOD: Nucleotide-binding oligomerization domain
CARD: Caspase recruitment domain
IOD: Integrated optical density value
